# When third-person perceptions motivate correction: political identity, anticipated guilt, and responses to political misinformation

**DOI:** 10.3389/fpsyg.2026.1814693

**Published:** 2026-08-04

**Authors:** Mihee Kim

**Affiliations:** Department of Media and Communication, Sejong University, Seoul, Republic of Korea

**Keywords:** third-person perception, political misinformation, partisan source, political identity, anticipated guilt, corrective actions

## Abstract

Drawing on the third-person effect (TPE), this study examines how partisan identity, source cues, and comparison targets shape third-person perceptions (TPP) of political misinformation and their associations with corrective intentions during the 2022 South Korean presidential election. The study further investigates anticipated guilt as a potential emotional mechanism associated with the relationship between partisan sources and corrective intentions. An online experiment (*N* = 479) showed that individuals exposed to counter-attitudinal misinformation exhibited stronger TPP than those exposed to pro-attitudinal misinformation, particularly when evaluating out-group rather than in-group targets. Partisan source effects emerged only among participants exposed to counter-attitudinal misinformation. Moreover, TPP involving out-group—but not in-group—targets was positively associated with anticipated guilt, which was in turn associated with stronger corrective intentions. Moderated serial mediation analyses further revealed that, among participants whose preferred candidate was targeted by misinformation, exposure to an out-group source was indirectly associated with stronger corrective intentions through TPP involving out-group targets and anticipated guilt. No corresponding indirect association was observed among participants exposed to misinformation targeting their non-preferred candidate. The implications of the findings are discussed.

## 1 Introduction

In highly polarized political environments, political misinformation is rarely evaluated in neutral terms; rather, it is interpreted through partisan identities (Kim, 2025; Tsang, 2022). Such identity-driven evaluations may shape not only perceptions of the influence of political misinformation but also individuals’ willingness to engage in corrective action. To explain these perceptual and behavioral responses, the present study draws on the third-person effect (TPE) (Davison, 1983). The TPE proposes that individuals tend to perceive media messages—particularly those regarded as undesirable or harmful—as exerting greater influence on others than on themselves (i.e., third-person perception [TPP]) (Perloff, 2009). The magnitude of TPP varies according to contextual and social factors, including the characteristics of comparison targets (Cohen et al., 1988; Meirick, 2004). Importantly, TPP has been linked to behavioral outcomes, including support for interventions intended to reduce the perceived societal harm of media messages (Kim, 2015; McLeod et al., 1997; Lo et al., 2015; Rojas, 2010).

Previous research suggests that perceived influence on others often predicts support for media regulation or censorship more strongly than the self–other perceptual gap (Gunther and Storey, 2003; Chung and Moon, 2016). However, the self–other disparity may become more consequential when outcomes involve personally relevant behavioral responses rather than general policy attitudes (Tewksbury et al., 2004). This distinction is particularly relevant in political misinformation contexts, where individuals may be motivated to intervene not simply because others are perceived to be influenced, but because certain audiences are perceived to be more vulnerable than themselves. Moreover, in polarized political environments, perceptions of vulnerability are likely to vary across partisan groups. Individuals may perceive politically like-minded others as relatively resistant to misinformation while viewing political opponents as especially susceptible. Thus, TPP provides a useful framework for understanding how judgments of relative vulnerability are associated with corrective responses to political misinformation.

Against this background, the present study examines how partisan identity and intergroup dynamics shape TPP of political misinformation and subsequent corrective intentions. Specifically, it investigates how candidate preference and partisan news sources (in-group vs. out-group) jointly shape TPP toward in-group and out-group comparison targets during the 2022 South Korean presidential election, in which liberal candidate Lee Jae-myung and conservative candidate Yoon Suk-yeol were locked in a closely contested race (Jung, 2022).

Although prior research has primarily conceptualized the relationship between TPP and behavioral responses as a direct cognitive process (Chung and Kim, 2021; Lee et al., 2023; Yang and Horning, 2020), relatively little attention has been devoted to the emotional processes through which perceptions of others’ vulnerability become associated with a felt obligation to act. Research on health misinformation suggests that perceiving others as vulnerable to misinformation can evoke anticipated guilt about failing to prevent harm, which is subsequently associated with corrective behavior (Sun et al., 2021, 2022). Building on this work, the present study examines anticipated guilt as a complementary emotional mechanism that may help explain how TPP is associated with corrective intentions in political misinformation contexts.

### 1.1 Candidate preference and TPP

The social desirability corollary suggests that the direction and magnitude of TPP depends on how socially desirable a message’s effects are perceived to be (Gunther, 1995; Jang and Kim, 2018). When media content is regarded as harmful or undesirable, individuals are more likely to believe that it affects others more than themselves (i.e., TPP), whereas socially desirable messages are more likely to elicit stronger perceptions of media influence on oneself than on others (i.e., the first-person effect) (Duck et al., 1995; 1998). Consistent with this account, prior research has shown that perceiving misinformation as socially undesirable is associated with stronger TPP (Jang and Kim, 2018; Luo et al., 2024).

This perceived social desirability is not an inherent property of messages but is socially constructed through political identity and intergroup dynamics (Kim, 2025; Lee and Cho, 2024). In political contexts, whether misinformation is perceived as undesirable often depends on its alignment with one’s partisan stance (Tsang, 2021, 2022). Motivated reasoning (Lodge and Taber, 2013; Lord et al., 1979) leads individuals to evaluate politically favorable misinformation as accurate, while dismissing claims that threatens their political identity (Lee, 2023; Lee and Jang, 2023; Meirick and Franklyn, 2022).

These perspectives suggest that misinformation attacking one’s preferred candidate is more likely to be perceived as socially undesirable and identity-threatening than misinformation targeting the opposing candidate. To protect their self-concept, individuals exposed to counter-attitudinal misinformation are therefore expected to distance themselves from its influence while attributing greater susceptibility to others, resulting in a stronger TPP. Accordingly, the following hypothesis is proposed:

*H1:* Individuals exposed to misinformation attacking their preferred candidate will exhibit a stronger TPP than those exposed to misinformation attacking the opposing candidate.

### 1.2 Candidate preference and comparison target

Research on the TPE consistently demonstrates the social distance corollary, whereby perceived media influence increases with the social and psychological distance of the comparison target from the self (Cohen et al., 1988; Perloff, 2009; Turner et al., 1987). According to self-categorization theory (SCT), when partisan identities become salient, individuals categorize themselves and others into in-group and out-group members, making intergroup distinctions psychologically meaningful (Lee and Kim, 2025; Reid, 2012; Reid et al., 2005; Reid and Hogg, 2005). Consequently, politically like-minded others are perceived as sharing one’s own resistance to misinformation, whereas political opponents are viewed as more susceptible to its influence (Campbell, 1958; Hogg and Reid, 2006; Tajfel and Turner, 1979). As a result, individuals tend to perceive relatively little influence of misinformation on in-group targets while attributing greater influence to out-group targets.

Prior research suggests that comparative perceptions are shaped not only by social distance but also by normative fit—the extent to which a particular audience is perceived as receptive to, or compatible with, a given message (Reid and Hogg, 2005; Reid et al., 2007). Applying this perspective to political misinformation, TPP of misinformation can depend on the extent to which the message is viewed as consistent with the attitudes and predispositions of a particular target group.

Specifically, when misinformation attacks an individual’s preferred candidate (counter-attitudinal misinformation), the message is likely to be perceived as more compatible with the attitudes of political opponents. Consequently, out-group members are expected to be viewed as especially susceptible to such misinformation, whereas in-group members are perceived as relatively resistant. This pattern can widen the perceived susceptibility gap between out-group and in-group targets.

By contrast, when misinformation attacks a non-preferred candidate (pro-attitudinal misinformation), the message may be viewed as more compatible with the attitudes of the in-group than the out-group. Under these circumstances, the message is no longer perceived as uniquely persuasive to political opponents. As a result, perceived differences in susceptibility between out-group and in-group targets can be reduced. Accordingly, the following hypothesis is proposed:

*H2:* Candidate preference and comparison target will interact in predicting TPP, such that TPP will be higher for out-group targets than for in-group targets when misinformation attacks an individual’s preferred candidate, whereas this difference will be reduced or absent when misinformation attacks a non-preferred candidate.

### 1.3 Candidate preference and partisan source

Because political misinformation is often ambiguous and difficult to verify, individuals frequently rely on source cues as heuristic indicators of credibility (Petty and Cacioppo,1986). In polarized political environments, however, evaluations of source credibility are filtered through partisan identities, leading individuals to trust ideologically congruent sources while distrusting incongruent ones (Reid and Hogg, 2005; Hornsey, 2008). Therefore, misinformation attributed to out-group sources is more likely to be perceived as deceptive and socially consequential.

The influence of partisan source cues is particularly pronounced when misinformation threatens an individual’s preferred candidate (Reid and Hogg, 2005; Kim, 2025; Tsang, 2021). Under these circumstances, source cues become particularly influential as individuals pay closer attention to the credibility and motives of the communicator. As a consequence, misinformation originating from an out-group source is expected to generate stronger TPP than misinformation from an in-group source. In contrast, when misinformation targets a disliked candidate, source cues are expected to play a smaller role because the message poses less identity threat. Accordingly, the following hypothesis is proposed:

*H3:* Candidate preference and partisan source will interact in predicting TPP, such that TPP will be higher for out-group sources than for in-group sources when misinformation attacks an individual’s preferred candidate, whereas this difference will be reduced or absent when misinformation attacks a non-preferred candidate.

Although prior research suggests that partisan source cues and candidate preferences jointly shape TPP, it remains unclear whether this interaction varies by the comparison target. Out-group comparisons may amplify this interactive effect through heightened intergroup contrast, whereas in-group comparisons may attenuate it due to perceived similarity (Lee, 2023; Lee and Cho, 2024). Accordingly, the present study asks:

*RQ1:* Does the interaction between candidate preference and partisan source on TPP differ by the comparison target (in-group vs. out-group)?

### 1.4 Anticipated guilt

According to cognitive appraisal theory (Lazarus, 1991; Roseman and Smith, 2001), individuals interpret situations based on their perceived implications for personal and social well-being, and these appraisals give rise to specific emotional responses. Within this framework, guilt arises when individuals appraise their actions or inaction as inconsistent with internalized moral values, particularly in situations in which they perceive personal responsibility and a degree of control over outcomes (Lazarus, 1991; O’Keefe, 2002). Such appraisals are not limited to retrospective evaluations but can also be prospective (Lindsey, 2005). Individuals may anticipate the emotional consequences of future choices and experience anticipated guilt when they foresee that failing to act could result in harm to others, thereby violating moral or civic obligations (Lindsey, 2005; Tangney et al., 2007). In this sense, anticipated guilt represents a forward-looking emotional response that functions as a moral signal, alerting individuals to the potential consequences of inaction and motivating preemptive behavioral regulation (Birkimer et al., 1993).

Within misinformation settings, perceived vulnerability of others serves as a key antecedent of anticipated guilt. Sun et al. (2022) found that greater perceived vulnerability of others to anti-vaccination misinformation was associated with stronger anticipated guilt. Because TPP reflects the perception that others are more susceptible to misinformation than oneself, individuals who experience stronger TPP may likewise anticipate greater guilt about failing to intervene. Accordingly, the following hypothesis is proposed:

*H4:* TPP of political misinformation will be positively associated with anticipated guilt.

### 1.5 Anticipated guilt and corrective action intentions

The negative state relief model posits that individuals are motivated to engage in prosocial or corrective actions to alleviate psychologically aversive emotions such as guilt (Baumann et al., 1981; Boster et al., 1999). Building on this perspective, when individuals anticipate future self-blame or regret for failing to act in accordance with moral norms, they become motivated to engage in behaviors that can prevent such negative self-evaluative emotions (O’Keefe, 2000; O’Keefe, 2002). In this way, anticipated guilt functions as a pre-behavioral motivational force shaping intentions rather than a *post hoc* emotional response (Lindsey, 2005). This theoretical account is supported by prior research demonstrating positive associations between anticipated guilt and a wide range of prosocial, preventive, and norm-compliant behaviors (Boudewyns et al., 2013; Elgaaied, 2012; Steenhaut and Van Kenhove, 2006; Wang, 2011). In misinformation contexts, particularly in public health domains, anticipated guilt has likewise been shown to motivate corrective engagement (Sun et al., 2021, 2022).

Although political misinformation may not pose immediate physical risks, it can generate serious societal harm by misleading voters, distorting public opinion, and undermining electoral integrity (Freelon and Wells, 2020). This suggests that guilt-based motivational processes similar to those observed in health misinformation contexts may also operate in political settings. Particularly in polarized political environments, individuals may view political misinformation as a threat to democratic outcomes and as a source of potential social harm. In such contexts, corrective action may function as a psychologically adaptive strategy, because people anticipate guilt about remaining passive and attempt to relieve this anticipated guilt by confronting false political claims. Accordingly, the present study proposes the following hypothesis:

*H5:* Anticipated guilt will be positively associated with individuals’ intentions to correct political misinformation on social media.

If the proposed hypotheses are supported, the observed associations may be consistent with a sequential pathway in which partisan source is associated with intentions for corrective actions through TPP and anticipated guilt. Because these associations may be more pronounced under conditions of identity threat, the present study further examines whether candidate preference moderates this sequential indirect pathway. Accordingly, the following research question is proposed:

*RQ2:* To what extent does candidate preference moderate the sequential indirect association between partisan source and corrective action intentions through TPP and anticipated guilt?

## 2 Method

### 2.1 Participants

The study employed a 2 (source: in-group vs. out-group) x 2 (candidate preference: Lee vs. Yoon supporters) x 2 (comparison target: in-group vs. out-group) experimental design. Candidate preference was not manipulated but measured, and the comparison target was a within-subject factor. Participants were recruited through a professional online survey company that maintains a nationwide panel of Korean adults. Quota sampling was employed to approximate the demographic distribution of the Korean population in terms of age, gender, and geographic region. Of the 672 respondents who completed the experiment, those who did not support either major candidate or failed to complete the survey were excluded. The final sample consisted of 479 participants (51.4% male; *M* age = 44.85, *SD* = 13.89), who were randomly assigned to one of two Facebook post stimuli.

### 2.2 Ethical statements

The study was conducted in accordance with the Declaration of Helsinki and received approval from the Institutional Review Board of Sejong University, Seoul, South Korea (SUIRB-HR-E-2022-007, February 11, 2022). The IRB waived the requirement for informed consent because the study posed minimal risk, did not involve the collection of personally identifiable information, and did not target vulnerable populations.

### 2.3 Stimuli

Two mock Facebook posts were created by manipulating partisan news sources: *Chosun Ilbo* (conservative) and *Hankyoreh* (liberal). Each post was designed to resemble the newspapers’ official Facebook pages and contained anti-Lee misinformation that had circulated prior to the election. The post alleged Lee Jae-myung’s association with a former gangster, accompanied by a photograph and descriptive text suggesting undue influence. The post was not explicitly identified as misinformation during exposure, allowing participants to respond to the content under conditions that more closely approximated naturalistic information processing. Following completion of the survey, participants were debriefed and informed that the post contained fabricated information created solely for research purposes.

### 2.4 Pretest measures

In order to assess candidate preference, participants were asked to indicate their support for a candidate in the forthcoming presidential election on a 7-point scale ranging from 1 (*strongly supporting Yoon*) to 7 (*strongly supporting Lee*). Following previous research on partisan identification, respondents who selected the midpoint of the scale (4), indicating no preference for either candidate, were excluded from the analysis. Participants who selected values of 1–3 were classified as Yoon supporters, whereas those who selected values of 5–7 were classified as Lee supporters. Based on this classification, the final sample consisted of 255 Yoon supporters (*M* = 1.65, *SD* = 0.82) and 224 Lee supporters (*M* = 6.84, *SD* = 0.36). The difference in candidate preference between the two groups was statistically significant, *t* (477) = −88.14, *p* < 0.001.

### 2.5 Posttest measures

#### 2.5.1 TPP

TPP was measured using two items adapted from prior studies (Hyun and Seo, 2021; Kim, 2015), assessing perceived influence of the Facebook post on oneself and on Lee/Yoon supporters, including perceived effects on voting decisions (1 = no influence at all, 5 = a great deal). Responses were averaged for each target (*α* = 0.82 for self; α = 0.84 for Lee supporters; α = 0.82 for Yoon supporters). Based on participants’ candidate preference, perceived influence on Lee/Yoon supporters was recoded as influence on in-group or out-group members. TPP was calculated by subtracting perceived self-influence from perceived influence on in-group and out-group targets, yielding two indices (in-group TPP: *M* = 0.54, *SD* = 1.16; out-group TPP: *M* = 0.68, *SD* = 1.77).

#### 2.5.2 Anticipated guilt

Anticipated guilt was measured with four items (“remorseful,” “guilty,” “sorry,” “bad”) assessing how participants would feel if they failed to correct the post (1 = not at all, 7 = extremely; *M* = 3.69, *SD* = 1.80, α = 0.97).

#### 2.5.3 Corrective action intentions

Three items measured intentions to correct the misinformation (e.g., commenting, messaging, sharing corrections) on 7-point scales (*M* = 2.90, *SD* = 1.18, α = 0.96).

#### 2.5.4 Control variables

In addition to demographic factors such as gender, age, education level, and income, social media use were measured as control variables. Participants’ frequency of social media use was gauged with a 7-point scale from 1 (*not at all*) to 7 (*very often*) (*M* = 6.21, *SD* = 1.19).

## 3 Results

### 3.1 Manipulation checks

Repeated-measures ANOVA confirmed that Chosun Ilbo was perceived as more conservative (*M* = 2.28, *SD* = 1.60) than Hankyoreh (*M* = 4.86, *SD* = 1.67), *F* (1, 478) = 545.64, *p* < 0.001, *partial η^2^* = 0.53. Participants associated *the Chosun Daily* with support for Yoon (*M* = 2.28, *SD* = 1.59) and *Hankyoreh* with support for Lee (*M* = 4.82, *SD* = 1.67), confirming successful source manipulation (Table 1).

**Table 1:** Correlations among the study variables.

Variable	1	2	3	4
1. TPP (In-group)	—			
2. TPP (Out-group)	.32**	—		
3. Anticipated guilt	.08	.28**	—	
4. Corrective intentions	.11*	.35**	.66**	—

*N* = 479. TPP, third-person perception. *p* < 0.05; **p < 0.01.

### 3.2 Hypothesis tests

A 2 (between-subject factor: Lee supporter vs. Yoon supporter) × 2 (within-subject factor: in-group vs. out-group target) mixed ANCOVA was run to test H1–H2. Candidate preference had a significant main effect on TPP, *F* (1, 471) = 87.44, *p* < 0.001, ηp^2^ = 0.16. Confirming H1, Lee supporters reported higher TPP (*M* = 1.12, *SE* = 0.07) than Yoon supporters (*M* = 0.16, *SE* = 0.07). The interaction between candidate preferences and comparison targets was significant, *F* (1, 471) = 86.78, *p* < 0.001, *partial η^2^* = 0.16. Among Lee supporters, TPP was substantially higher when evaluating out-group targets (*M* = 1.56, *SE* = 0.11) than in-group targets (*M* = 0.67, *SE* = 0.08). In contrast, Yoon supporters showed relatively low and similar levels of TPP toward both in-group (*M* = 0.42, *SE* = 0.07) and out-group targets (*M* = −0.10, *SE* = 0.11). Thus, H2 was supported.

A 2 (between-subject factor: Lee supporter vs. Yoon supporter) × 2 (between-subject factor: in-group vs. out-group source) × 2 (within-subject factor: in-group vs. out-group target) mixed ANCOVA was run to explore H3 and RQ1. The interaction between candidate preference and partisan source was significant, *F* (1, 469) = 6.11, *p* = 0.014, *partial η^2^* = 0.013. Only among Lee supporters, anti-Lee misinformation from an out-group source (*M* = 1.32, *SE* = 0.11) generated stronger TPP than from an in-group source (*M* = 0.93, *SE* = 0.10). In contrast, for Yoon supporters, TPP did not differ substantially between an out-group source (*M* = 0.21, *SE* = 0.09) and an in-group source (*M* = 0.11, *SE* = 0.09). The three-way interaction (candidate preference × source × comparison target) was not significant, *F* (1, 469) = 0.26, *p* = 0.61, *partial η^2^* = 0.001. None of the covariates were statistically significant in all analyses (*p* > 0.05).

**Figure 1:**
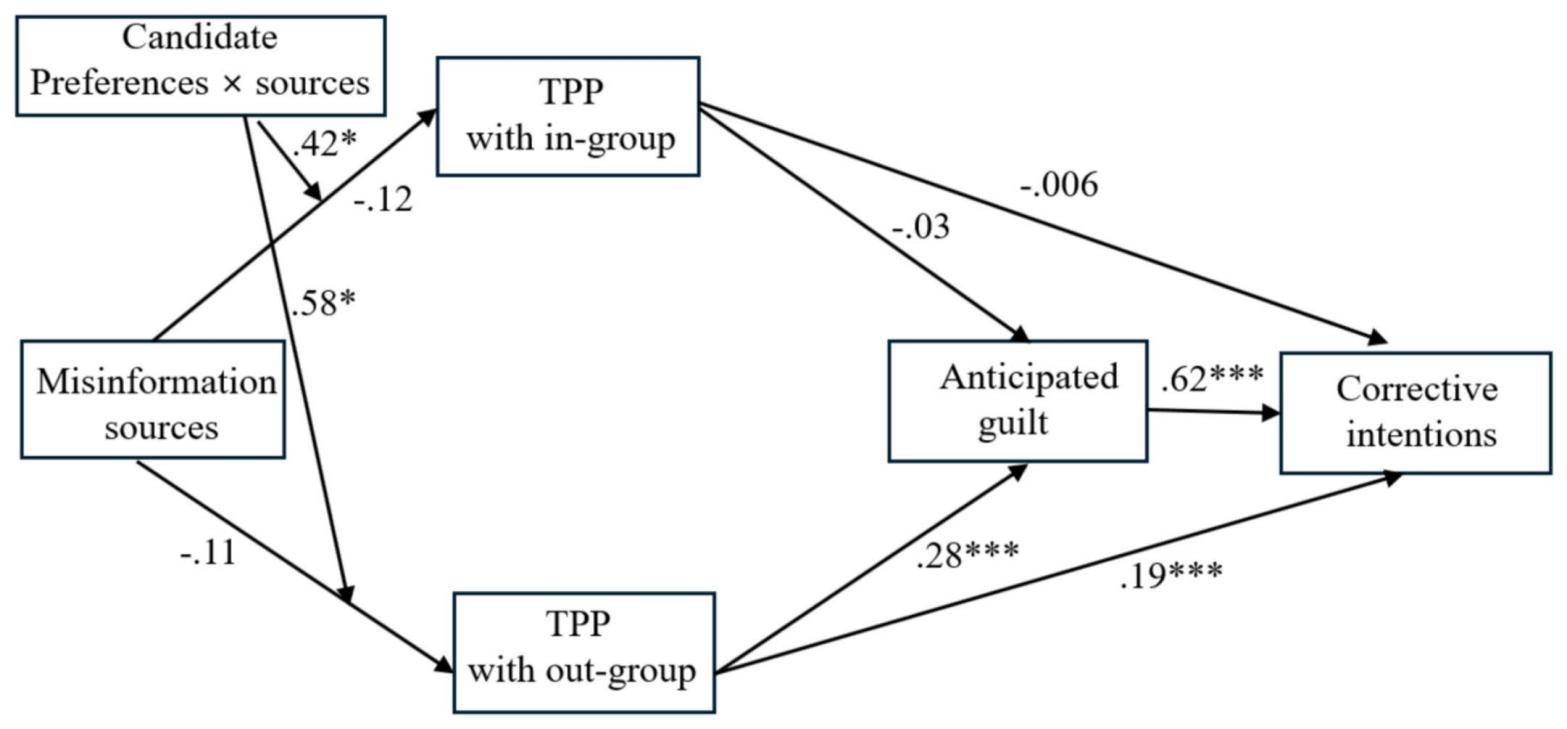
Moderated serial mediation model linking political misinformation to corrective intentions on social media. Candidate preference was coded as 0 = Yoon supporters and 1 = Lee supporters. News source was coded as 0 = in-group source and 1 = out-group source. Values represent unstandardized regression coefficients. TPP = third-person perception; × = interaction term; *p < 0.05; ***p < 0.001.

Lastly, a moderated serial mediation model was tested using the PROCESS macro (Hayes, 2022) to examine the relationships among the antecedents (source and candidate preference), TPP, anticipated guilt, and corrective intentions. A custom model was specified by adapting Model 81 to incorporate moderation (see Figure 1). Analyses employed 10,000 bootstrap samples with 95% bias-corrected confidence intervals, with all covariates statistically controlled. TPP compared with out-group targets significantly increased anticipated guilt (*b* = 0.28, *SE* = 0.05, *p* < 0.001), whereas TPP compared with in-group targets did not (*b* = −0.03, *SE* = 0.07, *p >* 0.05), Thus, H4 was partially supported. As predicted (H5), anticipated guilt was positively related to corrective intentions (*b* = 0.62, *SE* = 0.04, *p* < 0.001). Regarding RQ2, the index of moderated serial mediation was significant for the pathway through TPP involving out-group targets and anticipated guilt [*Index* = 0.10, *SE* = 0.05, *95% CI* = (0.001, 0.220)]. Specifically, only among participants whose preferred candidate was targeted by the misinformation [i.e., Lee supporters), exposure to an out-group news source was indirectly associated with stronger corrective intentions through increased TPP involving out-group targets and anticipated guilt (*b* = 0.08, *SE* = 0.05, *95% CI* = (0.001, 0.178)]. No corresponding conditional indirect effect was observed among Yoon supporters [*b* = −0.02, *SE* = 0.03, *95% CI* = (−0.084, 0.042)].

## 4 Discussion

This study examined how partisan identity, source cues, and comparison targets shape TPP of political misinformation and how these perceptions are associated with corrective action during the 2022 South Korean presidential election. The findings offer several implications for research on political misinformation and the TPE. First, the findings suggest that TPP of political misinformation cannot be explained by the social desirability of misinformation alone but is shaped by political identity and the meaning individuals attach to misinformation. Participants whose preferred candidate was targeted exhibited stronger TPP than those whose non-preferred candidate was targeted. Misinformation attacking a preferred candidate was likely perceived as more harmful and threatening, leading individuals to perceive greater influence on others than on themselves. By contrast, misinformation targeting a disliked candidate may have been interpreted as politically beneficial rather than socially harmful. Consequently, participants were less likely to perceive a large self–other disparity in media influence, resulting in weaker TPP.

The present findings suggest that perceived normative fit of political misinformation shapes TPP differently across comparison targets. As anticipated, Lee supporters exhibited substantially higher TPP for out-group than for in-group targets. They may have perceived anti-Lee misinformation as more compatible with the political predispositions of Yoon supporters (the out-group) than with those of fellow Lee supporters (the in-group). Consequently, political opponents were viewed as especially susceptible to the message, reinforcing the observed asymmetry in TPP. By contrast, Yoon supporters may have perceived anti-Lee misinformation as more compatible with politically aligned audiences (the in-group) than with committed Lee supporters (the out-group). This perception may have increased TPP involving in-group targets while simultaneously reducing TPP involving out-group targets. These opposing tendencies likely offset one another, resulting in relatively small differences in TPP between the two comparison targets.

A similar pattern emerged for partisan source cues. For Lee supporters, an out-group source was likely perceived as particularly effective at mobilizing audiences already predisposed against Lee or persuading undecided voters. In this context, the source, the message, and the presumed audience were normatively aligned, amplifying TPP. By contrast, Yoon supporters may have viewed an out-group source as having limited influence because it was presumed to reach primarily Lee supporters. An in-group source, however, was likely perceived as reaching politically aligned audiences, making the message appear more persuasive. These competing expectations may have offset one another, thereby reducing the influence of partisan source cues on TPP.

The moderated indirect findings further suggest that the motivational consequences of TPP depend on the political meaning attached to the TPP of misinformation. Only among Lee supporters, exposure to an out-group source indirectly associated with stronger corrective intentions through TPP involving out-group targets and anticipated guilt. Notably, TPP involving in-group targets was not associated with anticipated guilt. One possible explanation is that, even when Lee supporters perceived anti-Lee misinformation as influencing fellow supporters, they may have believed that it was unlikely to alter their existing political attitudes or voting intentions. Consequently, such influence may not have been perceived as sufficiently harmful to threaten electoral outcomes or evoke anticipated guilt. By contrast, TPP involving out-group targets likely carried greater political significance because anti-Lee misinformation was perceived as capable of reinforcing negative perceptions of Lee among political opponents and politically uncommitted voters, thereby reducing support for Lee and influencing electoral outcomes. This perceived political harm may have heightened anticipated guilt about failing to intervene, thereby strengthening corrective intentions.

No comparable indirect pathway emerged among Yoon supporters. Because the misinformation targeted an opposing candidate, its perceived influence may not have been interpreted as socially problematic. Instead, even when recognized as misinformation, it may have been viewed as politically advantageous by reinforcing negative attitudes toward Lee or increasing support for Yoon. Under these circumstances, TPP was less likely to evoke anticipated guilt or motivate corrective intentions. Taken together, these findings suggest that the behavioral consequences of TPP depend not simply on perceived audience vulnerability but on whether that perceived influence is interpreted as politically harmful or politically advantageous.

Several limitations should be acknowledged. First, the experimental stimuli consisted exclusively of misinformation attacking Lee. Consequently, Lee supporters were exposed to counter-attitudinal misinformation, whereas Yoon supporters were exposed to pro-attitudinal misinformation, confounding candidate preference with message congruency. Thus, the observed group differences may reflect not only message congruency but also broader ideological, psychological, or motivational differences between Lee and Yoon supporters. Second, although the proposed sequential pathway was theoretically derived, the mediator and outcome variables were measured concurrently. Therefore, the observed associations should not be interpreted as definitive evidence of causal mediation. Third, although participants were recruited from a nationwide panel, the sample was not nationally representative, limiting the generalizability of the findings. Finally, the study did not assess perceptions of the misinformation’s credibility or severity, both of which may have influenced TPP and anticipated guilt. In addition, although the anticipated guilt scale demonstrated high reliability, some items (e.g., *bad* and *sorry*) may capture broader negative affect rather than guilt specifically. Future research should employ more refined measures to distinguish guilt from related emotional responses.

Despite these limitations, this study demonstrates that TPP of political misinformation is driven by partisan identity and source cues. Crucially, the findings show that the emotional and behavioral consequences of TPP hinge on the political meaning attached to perceived media influence. By integrating partisan identity and anticipated guilt as key mechanisms, this study advances the literature on the TPE within polarized misinformation environments.

## Data Availability

The raw data supporting the conclusions of this article will be made available by the authors, without undue reservation.
